# Factors affecting private sector engagement in achieving universal health coverage: a scoping review

**DOI:** 10.1080/16549716.2024.2375672

**Published:** 2024-07-11

**Authors:** Naser Derakhshani, Ramin Rezapour, Saber Azami-Aghdash, Hamideh Nafar, Samira Soleimanpour, Emir Tahmazi Aghdam, Mohammadreza Maleki

**Affiliations:** a Health Management and Economics Research Canter, Health Management Research Institute, Iran University of Medical Sciences, Tehran, Iran; b Tabriz Health Services Management Research Center, Tabriz University of Medical Sciences, Tabriz, Iran; c Medical Philosophy and History Research Center, Tabriz University of Medical Sciences, Tabriz, Iran; d School of Health Management and Information Sciences, Iran University of Medical Sciences, Tehran, Iran

**Keywords:** Universal health coverage, private sector, engagement, scoping review, barriers, facilitators

## Abstract

**Background:**

Universal Health Coverage (UHC) is one of the most important strategies adopted by countries in achieving goals of sustainable development. To achieve UHC, the governments need the engagement of the private sector.

**Objective:**

The aim of this study was to identify factors affecting private sector engagement in achieving universal health coverage.

**Methods:**

The study is a scoping review that utilizes Arkesy & O’Malley frameworks. Data collection was conducted in MEDLINE, Web of Sciences, Embase, ProQuest, SID, and MagIran databases and the Google Scholar search engine. Also, manual searches of journals and websites, reference checks, and grey literature searches were done using specific keywords. To manage and screen the studies, EndNote X8 software was used. Data extraction and analysis was done by two members of the research team, independently and using content analysis.

**Results:**

According to the results, 43 studies out of 588 studies were included. Most of the studies were international (18 studies). Extracted data were divided into four main categories: challenges, barriers, facilitators, goals, and reasons for engagement. After exclusion and integration of identified data, these categories were classified in the following manner: barriers and challenges with 59 items and in 13 categories, facilitators in 50 items and 9 categories, reasons with 30 items, and in 5 categories and goals with 24 items and 6 categories.

**Conclusion:**

Utilizing the experience of different countries, challenges and barriers, facilitators, reasons, and goals were analyzed and classified. This investigation can be used to develop the engagement of the private sector and organizational synergy in achieving UHC by policymakers and planners.

## Paper context

- **Main findings:** Governments are key in healthcare provision, but the private sector’s involvement is increasingly vital for universal health coverage.
- **Added knowledge:** This paper explores the evolving role of the private sector in universal health coverage, analysing barriers, challenges, facilitators, reasons, and goals for engagement while suggesting areas for further exploration.
- **Global health impact for policy and action:** The private sector’s contributions to achieving Universal Health Coverage necessitate comprehensive policy frameworks and targeted actions to ensure equitable and sustainable health outcomes worldwide.

## Background

Universal Health Coverage (UHC) is the main policy that is greatly appreciated in the promotion of public health in different countries. World Health Organization (WHO) guidelines and instructions emphasize achieving this goal. UHC ensures that everyone can access the full spectrum of quality health services they need, whenever and wherever they need them, without facing financial difficulties. It encompasses the entire range of essential health services, including health promotion, prevention, treatment, rehabilitation, and palliative care throughout a person’s life [1–3]. WHO has introduced it as a strategy in countries to achieve fair health services and ultimately healthy society [4,5]. On 18 October 2019 the United Nations (UN) General Assembly’s 74^th^ session emphasized the importance of UHC in a political statement called ‘UHC: A collective movement toward a healthier world’ and recognized health and its dimensions as a prerequisite for sustainable development up to 2030 [6].

The implementation of UHC varies among countries due to differences in infrastructure, economic conditions, health policies, and social factors. Each country must develop a customized plan that is tailored to its unique circumstances and available resources. Political commitment and the healthcare workforce competency also play critical roles in shaping UHC strategies. Consequently, no single model for UHC fits all countries, requiring tailored approaches for effective implementation [7]. Based on special situations it may be necessary to take unique and different actions [7–10]. These unique features of countries have made UHC one of the most challenging political processes that need support and engagement on behalf of stakeholders such as health policymakers and the private sector [7]. For this reason, in UN statement (2019) in New York (UHC 2030) and articles 41, 45,50,53,54, and 80, it directly emphasized the importance and multilateral nature of acting in UHC and especially the engagement of the private sector in achieving UHC [11].

Today, one of the main and effective ways of facing health system challenges in all countries is utilizing private sector engagement and private–public partnership which can be called a multilateral and win–win policy. This engagement synergizes the capabilities of both parties in achieving their mutual goals [12,13]. It makes the private sector one of the most important and notable health service providers in low and middle-income countries (LMICs) [14,15].

The private sector can be a solution and a good opportunity for the growth of the health industry by providing facilities and innovative management and supporting the health system in achieving UHC [12,16]. Governments cannot ignore the role of the private sector in achieving UHC for their numerous activities in the health system [17,18]. Even the governments look at the private sector as a key, efficient, and cost-effective mechanism in the implementation of their goals and policies [18,19].

One of the reasons that goals such as justice and quality in UHC are threatened in many countries is the loose management and indifference to the private sector [20]. The private sector only looks for its benefits in countries and is indifferent to the policies and goals of the health system. The reason for this indifference is that the management of health systems in these countries is not designed in a uniform and integrated form [20,21]. Moreover, in these countries, of private sector’s role and position in the health system are unclear and there is no plan for the collaboration and implementation of governments’ health policies in the private sector [17].

Achieving UHC is a vital step toward ensuring equitable access to basic health services for all populations. The private sector’s engagement with this initiative is critical, as it has the potential to substantially enhance healthcare systems’ capacity, efficiency, and accessibility. However, the integration of private sector into UHC efforts is often met with numerous challenges, including regulatory barriers, financial constraints, and differing operational priorities. Understanding these barriers, as well as the facilitators and motivations for private sector involvement, is essential for formulating effective strategies that foster collaboration and leverage private sector resources. Therefore, this study was conducted to identify factors affecting private sector engagement in achieving universal health coverage.

## Methods

### Design

The present scoping review was conducted in 2021. Arkesy & O’Malley framework was utilized. This was the cognitive methodological framework that guided the scoping review, which was published in 2005. This framework consists of six steps: identifying the research question, identifying related studies, selecting/screening studies, categorizing/dividing data, summarizing, synthesizing, and reporting results, and providing operational guidelines and recommendations. The Arksey and O’Malley framework is a clear, adaptable, and systematic approach to scoping reviews and also, it allows for refinement of research questions and search strategies, ensuring comprehensive and relevant findings [22].

### Data sources

First, through preliminary review and asking for comments from two experts and one medical librarian, the keywords were specified and a search strategy was designed and implemented. The keywords were as follows: ‘Universal Health Coverage’, ‘Universal Coverage’, ‘Universal Healthcare Coverage’, ‘Universal Health Care Coverage’, UHC, ‘Private sector’, ‘Private health sector’, ‘Private provider’, ‘Private-for-profit providers’, ‘Private-not-for-profit providers’, ‘Non-state providers’, ‘Public-private mix’, ‘Private institutions’, ‘Private actors’, ‘Non-governmental organizations’, Cooperation, Collaboration, Participation, Partnership, Interaction, Engagement, Contrib*, Involvement. Search was done in MEDLINE, Scopus, Web of Knowledge, Embase, ProQuest, SID, MagIran databases, and Google Scholar. The search time was 2008–2021. A manual search of several relevant journals and references of articles was conducted. Also, the official websites of organizations including WHO, World Bank (WB), and others were searched. To search for grey literature, we searched the Health Care Management Information Consortium, (HMIC), System for Information on Grey Literature in Europe (SIGLE), and European Association for Grey Literature Exploitation (EAGLE). (Search strategy supplementary file 1)

### Inclusion criteria

- Studies reporting the participation of the private sector in achieving UHC
- Studies between 2008-2021

### Exclusion criteria

- Studies reporting public–private partnerships in other fields and outside health system
- Studies that evaluated public–private partnerships with aims other than UHC
- Studies that limited public–private partnerships in the health system only to outsourcing and a mere contract between two parties
- Abstracts or congress abstracts without full text
- Articles that did not report enough about the subject

### Review process

The selection and screening process was conducted by two members of the research team, independently. The controversial items were solved through discussion and if needed were referred to the third person who was more knowledgeable and experienced. At first, the titles were evaluated and irrelevant studies were excluded. Then, the abstracts and full texts were evaluated to identify irrelevant studies. Endnote X8 software was utilized to evaluate titles, abstracts, and repeated items. To report selection and screening results PRISMA flowchart was utilized [23–25].

### Quality appraisal

Due to the condition of the scoping review, quality appraisal of reviewed sources was not conducted [22].

### Data extraction

To extract data according to the objectives and based on preliminary evaluation, the extraction form was designed by Microsoft Word 2016. At first, five papers data were extracted for pilot study and the problems in preliminary form were solved. Two researchers independently extracted data from the selected articles and solved the ambiguities by consulting with research team members. Extracted data included: the name of the author and publication year, country, objective(s), the type of private sector, activity, challenges and barriers, facilitators, reasons and goal of engagement, and the results of engagement (pros and cons).

### Data analysis

The content analysis was applied in this study. This is a common method for identifying, analyzing, and reporting the patterns within the texts [26–29]. Coding was done by two researchers, independently. Steps of the analysis included: reading the text several times, getting familiarized with data, identifying and extracting the primary codes, identifying the themes by placing similar codes together, revising the themes, naming and defining the themes, and ensuring the reliability of identified codes and themes by reaching agreement between the two coders and resolving the disputes through discussion.

## Results

**Figure 1.**
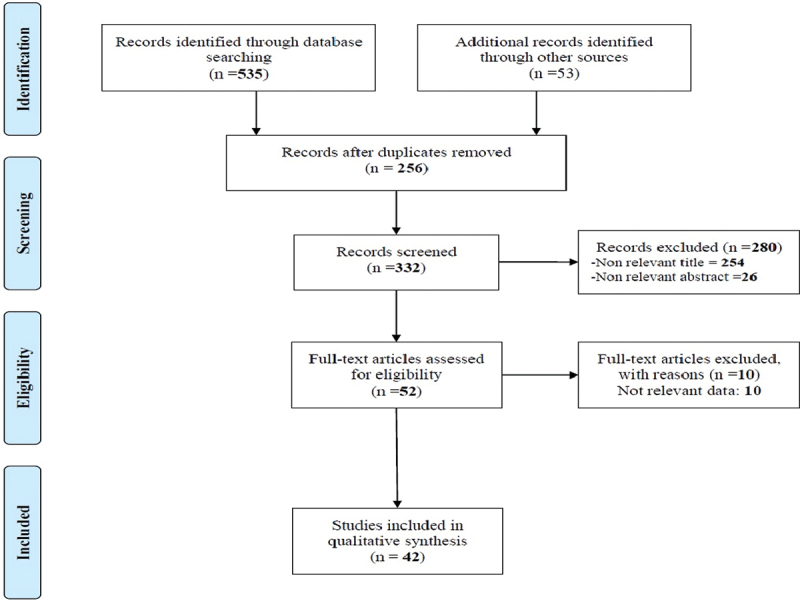
Flow diagram of the searches and inclusion process.

Out of 588 articles found through systematic and manual searches in databases and websites, 256 papers were excluded for repetition. Also, by screening titles and abstracts 280 articles were excluded for irrelevancy. Out of the remaining 52 articles, 10 were excluded for lack of enough data and not reporting the engagement of the private sector in achieving UHC. Finally, 42 eligible papers were entered into the study (Figure 1). The Supplementary file shows the summary of extracted data [14,18,30–69] (Supplementary file 2).

**Figure 2.**
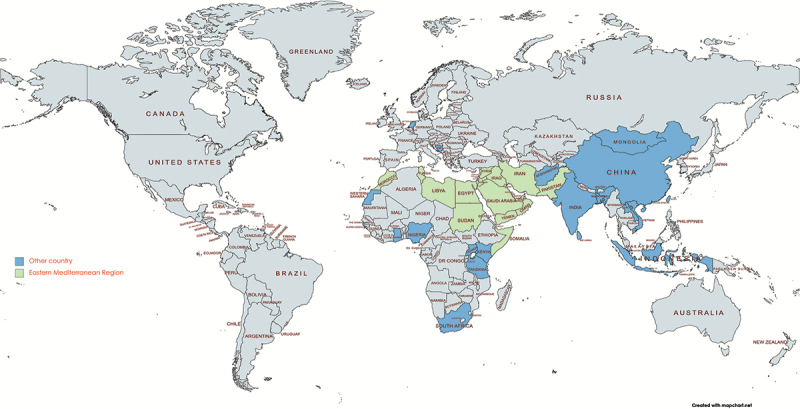
Studies in countries evaluating private sector participation in achieving UHC.

The studies were conducted in 20 countries with most of them to be carried out in India (5 cases). Asia and Africa had the highest number of included studies. Out of 42 articles, 16 were international and two were in the WHO Eastern Mediterranean Region (Figure 2).

**Figure 3.**
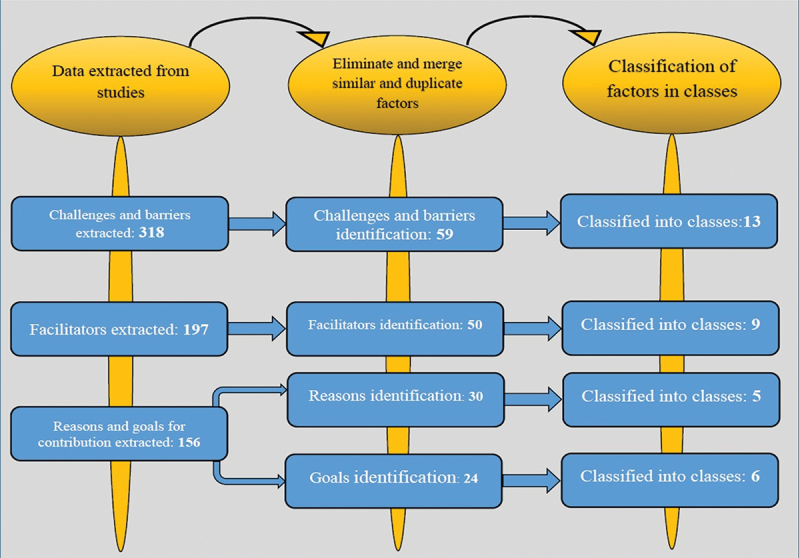
The process of identifying, screening, selection, and categorization of factors.

According to data analysis, the extracted data were classified into four main categories barriers and challenges, facilitators, reasons for engagement, and goals of engagement (Figure 3).

### Barriers and challenges of private sector engagement in achieving UHC

Initially, 318 barriers and challenges were identified. After the exclusion of repetitions and integration of homogenous and similar items, 59 challenges and barriers were classified under 13 categories. Regarding the frequency of barriers and challenges, economic challenges (nine items) and organizational challenges (eight items) were the most frequent categories (Table 1).

**Table 1.** Challenges and barriers to private sector engagement in achieving UHC.

Main Themes	Sub-Thems
Quality Challenges	The uneven quality of private health systems compared to each other Concerns about the low-quality of healthcare services provided by private sectors Weak mechanisms for monitoring the quality of health services
Regulations Challenges	Weak regulatory frameworks Weaknesses in implementing regulations Poor at following national guidelines Lack of standardization in the private sector
Resource Barriers	Waste of resources in public and private sectors The private sector Undermines allocative efficiency Unequal distribution resource of health care system
Accreditation and Qualified Challenges	Under-qualified private providers The high cost of licensing and lack of qualifications to apply Longtime Process of Accreditation and licensing requirements for the private sector Poor or limited support in providing occupancy certificates for the private sector
Human resources Challenges	Weakness in attracting and retaining health workers in non-state providers Brain drains from the public sector High staff turnover in non-state providers Overreliance on public sector-trained staff
Ethical Barrier	Corruption in public and private sectors when working together Unresolved conflicts of interest in the public and private sector Unethical practices (induced demand: Over-servicing with private hospitals)
Financial Challenges	Lack of Drawing up healthcare benefit packages and flexibility for the private sector Districts’ financial dependence on donor funds channeled through the central government limited their actual autonomy for establishing contracts directly with NSPs. High administrative costs in the public sector for contracting out with the private sector Delays in reimbursements to the private sector through the public sector High Out-of-pocket payment in the private sector Uneven regional economic development Low levels of government salary for health workers Funding insecurity in public and private sectors when working together Purchasing poor-value care from private sector
Governance Challenges	Weak governance mechanism Inappropriate political interference in key decisions Weakness of government policies in the health sector Weak stewardship health system The role of the private health sector is not well defined in achieving universal health coverage Mistrust between the public and private sectors Limited inter-sectoral collaboration between the public and private sectors
Organizational Barrier	Mismatched organizational styles and differing priorities in public and private sectors Fragile program due to use of donor funding for embarking on public–private partnerships Dual practice between the public and private sectors Poor forecasting and planning for patient load, resulted in NSPS complaining. Weak contract frameworks between the public and private sectors Lack of incentives in public sector A large private sector undermines the integrity and sustainability of the health system overall Imbalance of power between the private and public sectors
Cultural Barriers	Socio-cultural influences such as the need for female health care providers to serve female clients Normative gaps (clear and comprehensive standards and frameworks are lacking to guide a country’s efforts) Little community participation due to large private sector
Services delivery Challenges	Balance tilting towards curative care by private providers Heterogeneity in public and private providers Provides fragmented care in private sector
Market Barriers	Market condition barrier: abuse of market power (market skimming monopolistic behavior and predatory pricing) for profit private sector Limited government control of medicine promotion and advertising Protecting equity
Information Challenges	Private providers do not report to the ministry of health for fear of increased taxation Management information systems were inadequate to monitor the performance of private providers Gaps in evidence (Poorly documented) to decision-making Lack of common understanding Weakness of the health research systems

Achieving UHC faces numerous challenges and barriers across various axes. Quality challenges include poor and inconsistent private sector services, exacerbated by inadequate monitoring. Regulatory issues stem from weak frameworks, poor execution, non-compliance with national guidelines, and lack of standardization in the private sector. Resource barriers involve inefficient use and unequal distribution of resources in both public and private sectors, undermining allocative efficiency. Also, human resource issues are characterized by difficulties in recruiting and retaining professionals, brain drain from the public sector to the private sector, high turnover rates, and overreliance on public sector-trained staff. Accreditation challenges include low-quality providers, high costs, lengthy procedures, and insufficient support for occupancy permits. Ethical hurdles involve corruption, conflicts of interest, and unethical practices like induced demand in private hospitals.

Financial barriers include inflexible healthcare plans, reliance on donor funding, high administrative costs, delayed reimbursements, high out-of-pocket expenses, low government salaries, unstable funding, and poor-quality care from providers. Governance challenges encompass weak processes, political interference, inadequate health policies, poor stewardship, unclear roles for the private sector in UHC, mistrust between sectors, and limited intersectoral collaboration. Organizational barriers include mismatched styles, dual-practice issues, poor patient load forecasting, weak contracts, lack of incentives, and power imbalances. Cultural barriers involve sociocultural influences, normative gaps, and low community participation. Service delivery challenges include heterogeneity, fragmented care, and a focus on curative care. Market barriers involve market power abuse, limited control over promotion, and equity protection. Information challenges include inadequate management systems, gaps in evidence and research, and poor reporting to health ministries.

### Facilitators of private sector engagement in achieving UHC

Initially, 197 facilitators were extracted. After the exclusion of repeated items and integration of similarities, 50 facilitators remained. The identified facilitators were classified under nine categories. Capacity building (10 items) and economic facilitators (9 items) were the most frequent facilitators. Contracts (two items) were the less frequent category (Table 2).

**Table 2.** Facilitators for private sector engagement in achieving UHC.

Main Themes	Sub-Thems
Regulation Facilitators	Pay attention to economic regulation due to regulation of monopoly and competition in the health sector Pay attention to social regulation to achieve and promote social objectives. The improvement of regulatory frameworks through, quality accreditation and standards of care Regulate by the government on how to services delivery in the private sector Effective regulation of competition and management of the private sector contribution Strengthening government capacity to develop, implement, and monitor legislation and other regulations Establishing an appropriate system of incentives and penalties for more private involvement in complementing the government’s responsibility
Financial Facilitators	Increasing the Government’s adequate and sustainable financing for health systems cooperation with health private sector The review of fiscal policy (such as tariffs and import duties) Use of Technical and financial support from donors Develop strategies to limit operational costs in the private-not-for-profit sector Offering standardized service packages and prices that apply to all socioeconomic levels Voucher scheme financing to reduce financial barriers Subsidized to increase access to providers of reasonable quality Attention to Price determination bodies Effective strategic purchasing
Capacity Building	The capacity for management in the public sector to partner with the private sector The awareness and acceptability of structural changes related to responsibility and decisions (power and authority) for initiating commitment in a partnership Active involvement of the private health sector in national health strategic plans and establishment of formal dialogue mechanisms Using of tools of government for indirect governance of the private health sector Governments should take the lead and formulate domestic health goals and priorities The engagement of the private sector, beyond health, in improving access to health services Provision of the framework to guidance to bolster private sector participation International cooperation based on mutual learning across countries Culture shift to collaborate between public and private health sector Create a competitive environment between the big companies and local and small private providers
Policy Facilitators	Time and political commitment in initiating partnerships between the public and private sectors Having the political will to the management of the private sector contribution to ensure that coverage of basic health care There is a strong need for policy modifications to help boost private sector confidence and expand their participation in healthcare National policies to better distribute facilities geographically for better access. Adherence to policy leaving no one behind Develop a policy framework, organizational systems, and financing strategies for engaging the private health sector Minimize political interference in contract implementation
Evidence Base Decision	Adherence to Evidence-based national health strategies and leadership across both public and private sectors Knowledge and evidence on what works for health systems and UHC Public-private-partnerships need to ensure the appropriate use of basic signal functions
Monitoring	Developing comprehensive and elastic frameworks and tools for evaluating public–private partnerships Develop monitoring and reporting mechanisms Effective monitoring to clarify expectations and resolve any misunderstandings among the various stakeholders Assess the readiness of the public and private sectors to work together Independent quality assessment and Health care standardization in the private health sector All partners should be transparency and accountability for results to people and communities
Mobilize Human Resources	Assesses the availability of a properly trained workforce with the requisite numbers of staff Efforts by public private sectors to overcome workforce training. Recognize and leverage the PNFP sector’s extensive network of infrastructure and human resources.
Facilitate Contracts	Properly managing relationships between all actors to contract success Contract flexibility between the public and private sectors
Information Facilitators	Health Research to generates timely and reliable health information Need to be Supported by databases for private-sector mapping Collect and analyze information about how many and which private providers willing to engage

Managing private sector involvement in healthcare requires robust regulation addressing social and economic goals and competition. Strengthening regulatory frameworks with quality accreditation and care standards is essential. Governments should regulate private health service delivery and implement rewards and sanctions to increase private sector participation. Enhancing the government’s capacity to develop, implement, and monitor regulations is crucial for effective engagement. Sustainable funding, donor support, and affordable service packages promote public-private cooperation. Implementing voucher programs, provider subsidies, pricing organizations, and strategic purchasing can reduce financial barriers.

Developing public sector capacity to manage private collaborations involves recognizing changes in authority and accountability, establishing formal communication channels, and involving the private sector in national health plans. Governments should lead in setting national health goals, using indirect governance tools, and collaborating with the private sector outside health to enhance access. Political commitment is needed to regulate the private sector’s role in ensuring universal healthcare access. National policies should focus on equitable facility distribution and inclusive guidelines. Establishing organizational structures, financial plans, and policy frameworks to include the private sector while minimizing political interference is crucial. Leadership, evidence-based decision-making, and comprehensive partnership assessment frameworks are key to improving healthcare outcomes.

### Reasons for the engagement of the private sector in achieving UHC

In this study, 156 reasons were extracted for the engagement of the private sector in achieving UHC. After the exclusion of repeated items and integration of similar items, 30 reasons were identified. They were classified under five categories. Loose health systems, financial support, and resources were the most frequent (Table 3).

**Table 3.** The reasons for private sector engagement in achieving UHC.

Main Themes	Sub-Thems	
Weak health system	Broken health care system Limit number and Weak public sector The gap in public-sector ambulatory services Fill the gaps in secondary and tertiary care provision Overburdening of public sector hospitals Supplement the public sector which was damaged and weakened during a conflict Need to Quickly scale up vertical health programs Poor quality of services in both sectors	
Financial	Weak accessibility and affordability to comprehensive health services in the public sector Rising costs for healthcare services and restricted governments’ financial capacity Higher out-of-pocket spending Lack of investments public sector in the health Dependency on external donors to finance health services Limited mechanisms for pooling risks, resources, and weak enforcement in the tax system Political instability and economic crisis Financial motivations and high profits in the private sector	
Recourse Barriers	Infrastructure Challenges	The lack of government capacity to deliver basic health services to marginalized populations and certain populations or geographic areas Lack of capacity to manage the distribution of private health facilities Insufficient number of public health facilities Poor diagnostic capacity facilities in rural areas Inadequate infrastructure and medical supplies
	Workforce Barriers	Shortage of human resources less skilled providers Shortage of qualified healthcare professionals in rural areas
Demographic Changing	Demographic shifts including ageing Population displacement Non-communicable disease burden increase	
Community Pressure	Patient demand for better health care The problem of health inequality Excessive use of private sector services by urban and rural populations	

The involvement of the private sector in achieving UHC is driven by several critical factors, primarily the weaknesses of the public health system. Public health infrastructure is often broken, with limited capabilities and gaps in service provision, leading to overburdened public sector hospitals. The private sector can rapidly scale up health programs and enhance service quality across both sectors, addressing these critical gaps. Rising healthcare costs, limited government financial capacity, higher out-of-pocket expenses, and a lack of public investment led to dependency on external donors. Political instability and economic crises exacerbate these financial challenges, while the private sector’s potential for high profits drives its involvement.

Resource and workforce barriers also highlight the need for private sector participation. Insufficient public health facilities and inadequate infrastructure and medical supplies further complicate these issues. Additionally, workforce shortages, less skilled providers, and a lack of qualified healthcare professionals in rural areas present significant challenges. Demographic changes, such as an aging population, population displacement, and a growing burden of non-communicable diseases, add to healthcare complexities. Community pressures, including patient demands for better healthcare and prevalent health inequalities, also drive the need for private sector involvement, as it can offer improved healthcare options and help reduce access and quality disparities.

### Goals of the engagement of private sector in achieving UHC

After the evaluation of studies, 82 goals were extracted. After the exclusion of repeated items and integration of similarities, 24 remained. They were classified under 6 categories. Empowering health systems and resource management were the most frequent categories (Table 4).

**Table 4.** The goals of private sector engagement in achieving UHC.

Main Themes	Sub-Thems
Health System Strengthening	Investment in both medical education and hospital development Complement and integrated with the local health system Inter-sectoral collaboration to engage in interventions that affect health Enhance health system capacity and sustainability Using private sector health services to Supplement ministry capacity
Financing	Improve the government’s commitment to financing healthcare delivery Designing remuneration packages as per need Purchase of private-sector health services Encouragement by international financial institutions to rely on private actors to decrease the burden on national budgets
Resources Management	Ensure geographic spread of health service providers’ facilities Harnessing resources Better allocate resources to the population Create additional and newer infrastructure Contributions to training health workers
Promoting Access	Expand access to higher-quality health services Expansion of SHI and extend the depth and height of coverage Improved coverage of national health programs
Facilitate Cooperation	Develop a framework for partnerships with the private sector in variable circumstances of the country Implementation of a market-oriented approach through collaborative mechanisms that enhance accountability Implementation of standard treatment guidelines Prioritizing and explicit objectives public–private partnership context and policies
Promotion Health Services	Regulation of the private healthcare sector Improve Patient safety Controlling unnecessary service delivery

The goals of private sector engagement in achieving UHC are aimed at enhancing various aspects of the health system. Strengthening health systems involves private sector investments in medical education and hospital development, which complement and integrate with local health systems. This fosters inter-sectoral collaboration to implement health-related interventions, enhancing the capacity and sustainability of health systems with private sector services supplementing governmental capabilities. Financial goals include improving the government’s commitment to healthcare financing by designing need-based benefit packages and purchasing private-sector health services.

Resource management by the private sector focuses on ensuring a geographic spread of health service providers, better resource allocation to the population, and creating new infrastructure. Promoting access aims to expand access to higher-quality health services and extend health insurance coverage, thereby improving population health coverage. The promotion of health services emphasizes regulating the private healthcare sector to improve patient safety, control unnecessary service delivery, and ensure quality care, maintaining high standards and aligning private sector operations with national health objectives.

## Discussion

This scoping review provides an overview of the challenges faced by the private sector in achieving UHC, as well as the facilitators, reasons, and goals driving their engagement in this effort. Challenges and barriers with 59 items were classified into 13 categories, facilitators with 50 items were classified into 9 categories, and reasons (30 items) and goals (24 items) were classified into 5 and 6 categories, respectively.

The private sector faces several challenges in contributing to achieving UHC. These challenges were identified according to the different countries experiences. One major challenge to effective engagement is the diversity and heterogeneity of private sector health service providers, which increases the workload for the public sector and complicates collaboration efforts [14,44,70]. Moreover, despite its advantages for the health system, a wide-scale private sector can be a threat to the integrity and sustainability of the health system if remains uncontrolled [59,63]. The reason for this threat is the tendency of 70% of people to use health services provided by the private sector which causes the public facilities to be less utilized and finally deviated from national strategic goals [71]. Governmental challenges in the engagement of the private sector in achieving UHC are other problems that greatly affect the national health system [36,53,63,64,66]. Facing governmental challenges and trying to manage and solve them can contribute to a safe setting and bring trust among the stakeholders of private sector engagement in achieving UHC [37,66]. Also, possessing a reformist perspective on behalf of governments about governmental challenges can improve health system loose management and decrease political and utilitarian interferences by using evidence-based policy making and promotion of health service administrative capabilities it increases the efficiency and synergy of public and private sectors in achieving national strategic goals [45,63,72]. Furthermore, by developing the capabilities in both public and private sectors to facilitate engagement and by its management, accurate implementation of the rules, eliminating ethical challenges in both public and private sectors in terms of conflicts of interest, and ensuring the quality of health services in both sectors, we can partially eliminate engagement challenges and accelerate countries achieving in UHC [36–38,41,42,45].

This study assessed the experiences of countries that identified the most effective facilitators and classified them under 9 categories. They can be used in developing national health strategic plans by policymakers and planners. One of the most important facilitators that can accelerate the engagement of the private sector in achieving UHC is the use of efficient databases to collect and analyze information about the private sector in their tendency to cooperate and how they can cooperate with the public sector [43,45,48,51,73]. Utilizing efficient databases, governments can identify and analyze the potentialities of the health private sector according to their needs and then fill the gaps in the public sector.

The development of the private sector in the health system and its increased effectiveness on public health has partially pushed the health system for marketing. This factor can have negative effects on the health system. One of the key facilitators that can play both a facilitator and an approach in avoiding health marketing and also propel and control private sector participation in achieving UHC is the regulations. Using regulations, the governments can adjust rules in the form of win–win games and control the private sector in achieving UHC [14,32,35,44,47]. The Governments can correctly use the regulation as facilitators if they can fully control and manipulate factors such as the quality and quantity of services, allocation of health resources, providing competitive settings managing private sector share, and improving supervision [50,64,74–77]. It is also necessary for governments to go beyond the control of the private sector and consider regulative, economic, political, social, and even organizational structures [67].

The governments consider the private sector engagement in the health system to achieve their strategic goals. In this scoping review, we extracted the reasons and goals of private sector engagement in achieving UHC and classified them, respectively, under five and six categories. The loose and impotent public sector in providing qualitative health services and guaranteeing health security in LMICs is the most important reason for their need to use private sector engagement in achieving UHC [30,43]. Moreover, the health system is highly loose in some countries due to the lack of health financial resources or insufficient financial allocations on behalf of the government and inappropriate distribution of resources. On the other hand, the need for appropriate and accessible facilities increases the need for the presence of the private sector in the health system [18,51]. For this reason, governments try to build an integrated and sustainable health system and provide their citizens with qualitative health services as a valuable goal. Therefore, governments cannot ignore the engagement of the private sector due to their own needs and goals [37,42].

## Limitation

However, as a limitation, the present study used one language (English) for the search and collection of data. While the studies on the engagement of the private sector in achieving UHC might be conducted in different countries with their native languages they have not been retrieved and evaluated in this study. Another limitation is that we narrowed the engagement of the private sector to the goal of achieving UHC; while in other fields of the health system, the public–private partnership might exist but not for achieving UHC.

## Conclusion

The results suggested that despite many efforts in countries and the private sector engagement in achieving UHC, still many challenges and barriers exist. The present study tried to identify and classify the challenges, barriers, facilitators, reasons, and goals of this engagement. This study can be used by policymakers and planners in developing national health plans to achieve UHC with the private sector engagement. Moreover, utilizing the present results can predict the barriers and challenges that might happen in achieving UHC and provide situation-based solutions for these barriers to policymakers. The review showed that the more the goals of intersectional engagement are clear the more the consensus over solving these challenges that brings multilateral trust and facilitates and accelerates achieving UHC.

## Supplementary Material

Supplementary file 1.docx

Supplementary file 2.docx
