# Supplementary material for: Factors affecting private sector engagement in achieving universal health coverage: a scoping review
Source: Glob Health Action. 2024 Jul 11;17(1):2375672. doi: 10.1080/16549716.2024.2375672 (PMC11249157; doi:10.1080/16549716.2024.2375672)
Supplement: Supplementary file 1.docx [file ZGHA_A_2375672_SM1947.docx]

| **Supplementary file 1**: Complete search strategy for MEDLINE databases | | |
| --- | --- | --- |
| **Set** | **Strategy** | **Results** |
| **#1** | (((("universal health coverage"[Title/Abstract]) OR ("universal healthcare coverage"[Title/Abstract])) OR ("universal health care coverage"[Title/Abstract])) OR ("universal coverage"[Title/Abstract])) OR (UHC[Title/Abstract]) | 4704 |
| **#2** | ((((((((("Private sector"[Title/Abstract]) OR ("Private health sector"[Title/Abstract])) OR ("Private provider"[Title/Abstract])) OR ("Private-for-profit providers"[Title/Abstract])) OR ("Private-not-for-profit providers"[Title/Abstract])) OR ("Non-state providers"[Title/Abstract])) OR ("Public-private mix"[Title/Abstract])) OR ("Private institutions"[Title/Abstract])) OR ("Private actors"[Title/Abstract])) OR ("Non-governmental organizations"[Title/Abstract]) | 10891 |
| **#3** | (((((((Cooperation[Title/Abstract]) OR (Collaboration[Title/Abstract])) OR (Participation[Title/Abstract])) OR (Partnership[Title/Abstract])) OR (Interaction[Title/Abstract])) OR (Engagement[Title/Abstract])) OR (Contrib*[Title/Abstract])) OR (Involvement[Title/Abstract]) | 2816639 |
| **#4** | #1 AND #2 AND #3 | 98 |

| **Supplementary file 1**: Complete search strategy for **Scopus** databases | | |
| --- | --- | --- |
| **Set** | **Strategy** | **Results** |
| **#1** | ( TITLE-ABS-KEY ( "Universal Health Coverage" ) OR TITLE-ABS-KEY ( "Universal Coverage" ) OR TITLE-ABS-KEY ( "Universal Healthcare Coverage" ) OR TITLE-ABS-KEY ( "Universal Health Care Coverage" ) OR TITLE-ABS-KEY ( uhc ) ) | 8281 |
| **#2** | ( TITLE-ABS-KEY ( "Private sector" ) OR TITLE-ABS-KEY ( "Private health sector" ) OR TITLE-ABS-KEY ( "Private provider" ) OR TITLE-ABS-KEY ( "Private-for-profit providers" ) OR TITLE-ABS-KEY ( "Private-not-for-profit providers" ) OR TITLE-ABS-KEY ( "Non-state providers" ) OR TITLE-ABS-KEY ( "Public-private mix" ) OR TITLE-ABS-KEY ( "Private institutions" ) OR TITLE-ABS-KEY ( "Private actors" ) OR TITLE-ABS-KEY ( "Non-governmental organizations" ) ) | 74202 |
| **#3** | ( TITLE-ABS-KEY ( cooperation ) OR TITLE-ABS-KEY ( collaboration ) OR TITLE-ABS-KEY ( participation ) OR TITLE-ABS-KEY ( partnership ) OR TITLE-ABS-KEY ( interaction ) OR TITLE-ABS-KEY ( engagement ) OR TITLE-ABS-KEY ( contrib* ) OR TITLE-ABS-KEY ( involvement ) ) | 8618849 |
| **#4** | #1 AND #2 AND #3 | 212 |

| **Supplementary file 1**: Complete search strategy for **Embase** databases | | |
| --- | --- | --- |
| **Set** | **Strategy** | **Results** |
| **#1** | 'universal health coverage':ab,ti OR 'universal coverage':ab,ti OR 'universal healthcare coverage':ab,ti OR 'universal health care coverage':ab,ti OR uhc:ab,ti | 5365 |
| **#2** | 'private sector':ab,ti OR 'private health sector':ab,ti OR 'private provider':ab,ti OR 'private-for-profit providers':ab,ti OR 'private-not-for-profit providers':ab,ti OR 'non-state providers':ab,ti OR 'public-private partnership':ab,ti OR 'private institutions':ab,ti OR 'private actors':ab,ti OR 'non-governmental organizations':ab,ti | 13458 |
| **#3** | cooperation:ab,ti OR collaboration:ab,ti OR participation:ab,ti OR partnership:ab,ti OR interaction:ab,ti OR engagement:ab,ti OR contrib*:ab,ti OR involvement:ab,ti | 3505664 |
| **#4** | #1 AND #2 AND #3 | 108 |

| **Supplementary file 1**: Complete search strategy for **web of sciences** databases | | |
| --- | --- | --- |
| **Set** | **Strategy** | **Results** |
| **#1** | TS=(“Universal Health Coverage”) OR TS=(“Universal Coverage”) OR TS=(“Universal Healthcare Coverage”) OR TS=(“Universal Health Care Coverage”) OR TS=(UHC) | 5318 |
| **#2** | TS=("Private sector") OR TS=("Private health sector") OR TS=("Private provider") OR TS=("Private-for-profit providers") OR TS=("Private-not-for-profit providers") OR TS=("Non-state providers") OR TS=("Public-private mix") OR TS=("Private institutions") OR TS=("Private actors") OR TS=("Non-governmental organizations") | 31920 |
| **#3** | TS=(Cooperation) OR TS=(Collaboration) OR TS=(Participation) OR TS=(Partnership) OR TS=(Interaction) OR TS=(Engagement) OR TS=(Contrib*) OR TS=(Involvement) | 6364341 |
| **#4** | #1 AND #2 AND #3 | 99 |

| **Supplementary file 1**: Complete search strategy for **ProQuest** databases | | |
| --- | --- | --- |
| **Set** | **Strategy** | **Results** |
| **#1** | ti("universal health coverage") OR ti("universal healthcare coverage") OR ti("universal health care coverage") OR ti("universal coverage") OR ti(UHC) | 349 |
| **#2** | ti("Private sector") OR ti("Private health sector") OR ti("Private health sector") OR ti("Private provider") OR ti("Private-for-profit providers") OR ti("Private-not-for-profit providers") OR ti("Non-state providers") OR ti("Public-private mix") OR ti("Private institutions") OR ti("Private actors") OR ti("Non-governmental organizations") | 71671 |
| **#3** | ti(Cooperation) OR ti(Collaboration) OR ti(Participation) OR ti(Partnership) OR ti(Interaction) OR ti(Engagement) OR ti(Contrib*) OR ti(Involvement) | 2212733 |
| **#4** | #1 AND #2 AND #3 | 3 |
