# Supplementary material for: Factors affecting private sector engagement in achieving universal health coverage: a scoping review
Source: Glob Health Action. 2024 Jul 11;17(1):2375672. doi: 10.1080/16549716.2024.2375672 (PMC11249157; doi:10.1080/16549716.2024.2375672)
Supplement: Supplementary file 2.docx [file ZGHA_A_2375672_SM1946.docx]

| **Supplementary file 2.**  Characterized of studies included and summarized in the study | | | | | | | | | | |
| --- | --- | --- | --- | --- | --- | --- | --- | --- | --- | --- |
| **Num** | **Author: year** | **Country** | **Aim of the study** | **Type of private sector** | **Experiences or area of Participation** | **Challenges/ Barriers** | **Facilitator** | **Reasons** | **Result** | |
|  |  |  |  |  |  |  |  |  | **Disadvantages** | **Advantages** |
| 1 | Ejughemre, U. J. 2014 (1) | Nigeria | Highlights the potential for the Nigerian government to scale up healthcare financing by leveraging private resources, innovations, and expertise while working to achieve the universal health coverage. | - Private sector | - Healthcare financing | - Concerns about uneven quality - Concerns about affordability of private-driven health systems | - Providing enabling policies, regulatory environment - Creating appropriate incentives for more private involvement in complementing government’s responsibility | - Strengthening the health system - Complementing the government’s commitment to financing healthcare delivery - Innovations and expertise - Poor health expenditure: total and per capita - The problem of health inequality - the high burden of diseases - rising costs for healthcare services - Affordability of private-driven health systems | **If private sector is unregulated:**   - Growth of private involvement in health system financing and strengthening health system could worsen inequities - Limit health outcomes and undermine efforts to improve national health coverage - Jeopardizing the economic well-being of health clients | - Complementing the national government in healthcare financing |
| 2 | Bijoya Roy, 2017 (2) | India | Aspiring for Universal Health  Coverage through Private Care | - The Profit private sector - Not-for-profit | - Contracting and strategic purchase of services | - Drawing up healthcare packages and flexible | - Create a competitive environment between the big companies and local and small private providers | - Excessive use of private sector services by urban and rural populations | - Fragmentation and fail to integration health care delivery - More complex referral across the public and private sector - Purchasing of services from the private provider has increased | - --- |
| 3 | Hallo De Wolf, A and  Toebes, B. 2016 (3) | Netherlands | This article focuses on the role of the private sector in the direct provision of health care, the supply of health care-related goods, and health care financing. | - Multi-National companies - Non-governmental organizations - Non-profit entities - Charitable bodies - Private individuals - Civil society organizations | - Direct provision of health care - The supply of health care-related goods - Health care financing - The management of health care institutions | - Weak regulatory frameworks - Difficulty of guaranteeing independent, transparent, and effective regulatory bodies | - Economic regulation - Social regulation - Regulatory measures that aim to steer or alter the behavior of private actors | - Lack of inefficiency - Quality in the provision of public health care - Increased costs - Reduced budgets for health care due to the financial crisis - Encouragement by international financial institutions to rely on private actors to decrease the burden on national budgets. | - In some cases, private sector participation has led to increased costs for patients. | - Led to improve performance in terms of access and equity - Removing financial barriers to health care and minimizing the financial risks implied by illness - Making better use of resources |
| 4 | Iyer, V. Et al. 2016 (4) | India | This study investigated the adequacy of basic and comprehensive emergency obstetric care (bemoc and cemoc) services through the public and private sectors with reference to the United Nations (UN) guidelines. | - Private sector | - Purchasing emergency obstetric services | - Most of private (80%) facilities are concentrated in the towns | - Ensure appropriate use of basic signal functions. - National policies to better distribute facilities geographically for better access. - Monitoring and evaluation guidelines for emergency obstetric care with indicators | - Elimination of direct payments for obstetric services for the poorest women - Strengthen emergency obstetric services | --- | - Comprehensive obstetric service delivery by the private sector 3 to 11 times more than recommended standards - Elimination direct payments for obstetric services for the poorest women |
| 5 | Iyer, V. Et al. 2017 (5) | India | This paper aims to study the characteristics of eligible private obstetricians who chose to enter into a partnership with the state government to increase access to intrapartum care to disadvantaged women under the CY PPP program. | - Private sector | - Health services delivery to | - Qualified private providers are reluctant to work rural parts - Public sector is unable to attract and adequately staff rural health facilities - Distribution of free emoc provision | - We need to know how many and which private providers willing to engage in such partnerships | - Develop a framework for partnerships with the private sector in variable circumstances of country. - Implementation a market-oriented approach through collaborative mechanisms that enhance accountability - Control caesarean rates, - Designing remuneration packages as per need - Deal with collective phenomena like en masse participation or non-participation in the program - Ensure geographic spread of participant facilities - Create trusting relations among providers, local regulators and the public | - -- | - Reduction in maternal and neonatal mortality among the beneficiaries - Reduction detrimental practices like participating private facilities treating only low-risk, uncomplicated cases, and referring the more complicated cases to public hospitals. |
| 6 | Cowley, P. Chu, A. 2019 (6) | China Vietnam  Lao PDR | This commentary summarizes the growth of private hospitals in these three countries. Aspects of this private hospital growth are then described according to some UHC attributes such as quality, accountability, equity, and efficiency. | - Private for-profit hospitals | - Health services delivery in hospitals | - Overtreatment with private hospital - Quality of private hospital - Private hospitals have a fundamental problem addressing equity - Less interest by private hospitals in ensuring adequate resources for public hospitals - Absence of significant regulation | - Government financial support - Having well-functioning government mechanisms - Business model regulation - Professional self-regulation - National health insurance | - The lack of sufficient capital | - Increased risk of financial catastrophe and impoverishment. | - Non-state hospitals demonstrate more flexibility in the use of their human resources and shorter average lengths of stay than state hospitals. - Improving efficiency and quality |
| 7 | Rao, K. D. Et al, 2018 (7) | Afghanistan  Bangladesh  Bosnia & Herzegovina  Ghana  South Africa  Tanzania  Uganda | This paper report on the main learnings on the process of engaging nsps from the experience of these seven countries. | - Non-state providers: - (NGO, for-profit private providers and faith based non-profit providers) | - Service delivery | - Recruiting and retaining health workers in rural or other underserved areas - Quality of health services provided by NSPS - Human resources - Weak capacity and low sense of ownership at the central government level - Political interference - The lack of female health workers - Poor forecasting and planning for patient load - Not preparing realistic budgets - Weak capacity for monitoring of contracts - Weak governance mechanism - Weak mechanisms of accountability - The lack of clear delineation in the roles and responsibilities of different administrative levels - Inadequate capacity of local governments to manage contracts - Divorcing the financial and monitoring roles | - Properly managing relationships - The relationship between the central and lower administrative levels in contract management - Government stewardship capacity for monitoring contractual performance | - Limited public sector capacity - Inability of public sector services to reach certain populations or geographic areas - The widespread presence of NSPS in the health sector   **Afghanistan:**   - Broken health care system - Low human resources for health - Dependency on external donors to finance health services - No government capacity to deliver health services   **Bangladesh:**   - The lack of local government capacity to deliver basic health services to marginalized populations   **Tanzania:**   - Unable to adequately penetrate rural and remote areas | - Central government’s role in monitoring contracts was considered inadequate - Non- following cost sharing guidelines | - Increase coverage of health services - Increasing accountability - Reduced need to raise funds through user fees to recover their costs. |
| 8 | Maluka, S. 2018 (8) | Tanzania | This paper reports on perceptions of stakeholders on contracting-out faith-based hospitals through service agreements (sas) to provide primary healthcare services in Tanzania | - Non-state providers(faith-based hospitals) | - Provide primary healthcare services in rural areas | - Shortage of financial resources - Shortage of human resources - Constant delays in disbursement of funds - Inadequate Transparency and Mistrust Among Parties - High administrative costs - Lack of sufficient providers for meaningful competition in many rural areas - Power of vested interests - Inadequate and Constant Delays in Financial Support - Lack of Adequate Contract Enforcement Mechanism | - Good monitoring - Building public sector capacity to work with the private sector - Development of skills to negotiate and oversee contracts - Training and continuous technical support | - Central government needs to play a greater role in monitoring the implementation of the SA | - Reducing access and equity in the use of healthcare for poorest | - Same or higher quality at lower cost - Improving access to primary healthcare services |
| 9 | Maluka, S. Et al 2018 (9) | Tanzania | This paper  Reports on the design and implementation of service agreements (SAS) between local governments and NSPS for the provision of primary health care services in Tanzania | - Non-state providers:   (faith-based organizations (FBOS), (NGOS), private for-profit providers and informal providers) | - Delivery of primary health care | - Delays in reimbursements - Limited financial and technical capacity of local government authorities - Lack of trust between the government and private part - High administrative costs - Lack of sufficient providers for meaningful competition in rural areas - Existing vested interests among the parties - Overall shortages of funds - Inadequate capacity of the contracted parties to implement the contracts - Inadequate capacity of human resources | - Sources of financial and human resources | - Quickly scale up vertical health programs - Concerns about the quality of health care services - Lack of adequate health care personnel in the public sector | - Contracting-out may result in further fragmentation of the health system, particularly in countries where monitoring is weak | - Improved access to health services |
| 10 | Olu, O. Et al, 2019 (10) | Africa | Community participation and private sector engagement are fundamental to achieving universal health coverage and health security in Africa | - Private sector | - Innovation - Technology - Financing | - Fragmented implementation of the interventions - Limited inter-sectoral collaboration - Inadequate resources - Weak health systems - Inadequate hire core capacities - Inadequate community engagement in epidemic preparedness and response | - Legal, policy and regulatory environments - Building trust between the private and public sectors through continuous dialogue - Planning, implementation and monitoring of health programs | - Public resources limit - Shortages of critical cadres of health workers - Poor access to safe and affordable medicines | - --- | - Innovations for health care delivery - Networks for extending service coverage - Access to medicines and other medical commodities - Financial protection - Reduced patient costs by half |
| 11 | Lu, J. F. R &.  Chiang, T. L. 2018 (11) | Taiwan | Developing an adequate supply of health services: Taiwan's path to  Universal Health Coverage | - Private sector | - Creating health infrastructure | - --- | - Efforts in engaging private investment - Devise appropriate policy for ameliorate distribution imbalance - Regulate on the service sector to improve equality in the distribution of health resources | - Investment in both medical education and hospital development - Health service sector development | - --- | - Building human resources - Investment in medical institutions and built large hospital - Enriched the medical resources available to satisfy demand - Intensified competition in the health service market |
| 12 | Ssennyonjo, A. Et al. 2018 (12) | Uganda | Government resource contributions to the  Private-not-for-profit sector in Uganda:  Evolution, adaptations and implications for  Universal health coverage | - Non-profit provider | - Primary health care grants - Service coverage | - Financial challenges - Mismatch between government expectations and the resource needs of the PNFP sector | - Policies to offset costs of or subsidize private service provision - Contributions of resources from government to PNFPS should be revisited - Financial allocations should be increased - Strategic purchasing arrangements - Develop strategies to limit operational costs - Recognize and leverage the PNFP sector’s extensive network of infrastructure and human resources. | - --- | - --- | - Increased professionalization - Improved management capacities - Expansion of their networks - Improving compliance with standards - Increased efficiency |
| 13 | Wong, E. L. Et al. 2015 (13) | Hong Kong | How shall we examine and learn about public-private partnerships  (PPPS) in the health sector? Realist evaluation of PPPS in Hong Kong | - For-profit corporate - Non-profit organization | - Health service delivery | - Imbalance of power between the private and public sectors - Constraints on the public sector's procurement system | - Building of trust - Clearly defined objectives and roles - Time commitment - Transparency and candid information, particularly in relation to risk and benefit - Contract flexibility - Technical assistance or financial incentive behind procedural arrangements - The awareness and acceptability of structural changes related to responsibility and decisions (power and authority) - Values that motivate partners and demonstrating commitment - Coherent policy-planning framework | - Mobilizing resources - Enhance health system capacity and sustainability | - --- | - --- |
| 14 | Wadge, H. Et al. 2017 (14) | Cross-country | How to harness the private sector for universal health coverage | - Private sector | - --- | - Inadequate regulation - Insufficient access for the poor - Increased risk of inappropriate treatment that maximizes provider profit - Overreliance on public sector trained staff - Financial barriers - Organizational barriers - Geographical barriers - Cultural barriers | - Assesses the availability of a properly trained workforce with the requisite numbers of staff | - Complementary, integrated with the local health system - Work on areas of common concern such as medical education or communicable disease strategies - Fill in the gaps in secondary and tertiary care provision |  | - Improve access - Raise standards of care - Contribute to the achievement of UHC |
| 15 | Mcpake, B &  Hanson, K. 2016 (15) | Cross-country | Managing the public–private mix to achieve universal health  Coverage | - Private sector   (public–private mix, NGO, faith-based providers) | - --- | - Not provide comprehensive universal care - Low-quality - Under-qualified providers - Heterogeneity in public and private providers - Difficulty of affordability of private hospital development at the national level for a middle-income population | - Choosing policies that will contribute to the performance of the system as a whole - Financial incentives policies - Subsidized to increase access to providers of reasonable quality and hence support universal health coverage - Effective strategic purchasing - Effective regulation by competition, and consequent management of the private sector contribution - Regulate to the service delivery - Political commitment | - Government capacity is severely limited - No political appetite for ensuring that public subsidies are directed to those most in need - Affordability | - Private sector neglects important public health services, particularly preventive and primitive care | - --- |
| 16 | Shroff, Z. C. Et al. 2018 (16) | Cross-country | Moving towards universal health coverage:  Engaging non-state providers | - Non-state providers | - Service delivery | - Difficult geography - Socio-cultural influences - Lack of basic security - High staff turnover - Financial dependence on donor funds - Delayed reimbursements - Inadequate administrative capacity especially at the local government level - Absence of a mechanism to resolve disagreements - Attracting and retaining health workers - Lack institutional capacity to manage NSP engagement - Inappropriate rules and regulations - Inappropriate political interference in key decisions - Quality of health services provided - Trusting relationships among all the contracted parties | - All available human resources for health, whether in the public or private sector, need to be engaged - Having in place well-defined and formal contract - Supportive political leadership - Technical and financial support from donors - Strengthening capacity within both governments and nsps to develop and manage contracts - Effective monitoring - Ongoing communication to clarify expectations and resolve any misunderstandings among the various stakeholders. - Providing information - Establishing a system of incentives and penalties - Sufficient human, financial, monitoring and administrative capacity - Government stewardship capacity - Enforce regulation - Minimize political interference in contract implementation - Building institutional capacity to manage NSP engagement | - Delivering quality health services - Limited government enforcement capacity - Weak capacity - Shortage of human resources | - --- | - Strengthen health systems |
| 17 | Nabyonga. O. J, et al. 2019 (17) | Sub-  Saharan Africa | Partnership with private for-profit sector for universal health coverage in sub-Saharan Africa: opportunities and caveats | - Private for-profit sector | - Delivery of health services | - Weak Quality of services - Weak Pricing policies and the varying capacities - The dispensation that is based on client affordability rather than dosage requirement - Unethical practices - Proliferation of private providers - Minimal Participation of PFP in diseases outbreaks - Minimal Participation in preventive services | - Institutions Capacity for management - Culture to collaborate - Employing innovation | - Lack capacity to manage the distribution of private health facilities - less skilled providers - inadequate infrastructure and medical supplies - poor diagnostic capacity characterize facilities in rural areas | - --- | - Reduction in disparity in access between rich and poor, as well as urban and rural populations - Flexible to ensure timely access - Render services at times when public health facilities are closed |
| 18 | Morgan, R. Et al, 2016 (18) | Cross-Country | Performance of private sector health care: implications for universal health coverage | - Private sector (not-for-profit Provider & for-profit providers) | - --- | - Heterogeneity of the private sector - Low quality - unqualified providers - Lack of effective regulation - Use of potentially unnecessary and expensive procedures, such as caesarean sections - Weak public health system - Low efficiency of public expenditure - Lack of capacity to provide services - Dual practice - Burden of out-of-pocket expenditure - Lack of evidence - Lack of research and the difficulties of researching system-level | - Require a regulatory response focused on the health-care sector - Accreditation - Interactions | - Weak public sector - Patient demand for health care - Regulation of the private health-care sector - Individual factors - Poor performance and lack of availability of the public sector - Shortage of qualified health-care professionals in rural areas - Lower quality of care in the public sector | - Exclude poorer patients - Restricting private sector involvement mainly to higher quality services for richer people - Increase referral to the private sector - Increased patient costs - Increased absenteeism in the public sector - High-quality private sector that becomes inaccessible to lower socioeconomic groups | - Improving overall sector performance and population health |
| 19 | Ota, M. O. C. Et al, 2018 (19) | Africa | Proceedings of the first African Health  Forum: effective partnerships and  Inter-sectoral collaborations are critical for  Attainment of Universal Health Coverage in  Africa | - Private sector | - Digital financing - Health care service expansion | - Weakness of the health research systems - Weak research capacity - Poor research coordination - Duplication of efforts - Waste of resources - Inability to detect and track research and innovations | - Research for health - Regulatory frameworks - Accreditation systems | - Limited government resources - Inter-sectoral collaboration to engage in interventions that affect health | - -- | - Maximize strategic areas - Efficiency - Innovation - Capacity development - Expansion of health care services coverage - Health financing - Equity |
| 20 | Kumar, R. 2019 (20) | Sri Lanka | Public–private partnerships for universal health coverage? The future of “free health”  In Sri Lanka | - Private sector | - Health-service delivery | - Rising out-of-pocket expenditures - Poorly developed health information systems - Accreditation and licensing requirements - Weak private sector regulation - Weak mechanisms of accountability | - Regulation of Private Providers | - Gap in public-sector ambulatory services - Inadequate grossly investment in the public system - Expand access to higher-quality health services - Managerial capacity and knowhow from the private sector - Strengthening primary healthcare - Providing access to an essential-services package | - Intensify human resource deficits in the public sector | - --- |
| 21 | Suchman, L. Et al 2018 (21) | Ghana Kenya | Public–private partnerships in practice:  Collaborating to improve health finance  Policy in Ghana and Kenya | - Private sector   (NGO) | - --- | - Lack of information sharing - Weaknesses in management capacity - Funding insecurity - Mismatched organizational styles and differing priorities - Corruption | - Developing protocols for interaction - Ongoing, structured communication - Aggregating franchised private providers to facilitate interactions with government | - Health system weaknesses - Inadequate staffing of health facilities - Lack of basic medicine and health supplies - Insufficient number of public health facilities | - --- | - --- |
| 22 | Tsevelvaanchig, U. Et al 2018 (22) | Mongolia | This study examine regulatory architecture for private healthcare in Mongolia exploring its role for improving accessibility, affordability, and quality of private care and identifies gaps in policy design and implementation | - For-profit private | - Health-service delivery | - Do not exist important regulatory functions to quality of care at the national level - Poor quality - High cost of private care for the poor - Unequal distribution of private providers - High out‐of‐pocket - Lack of transparency and accountability in regulation - Lack of technical and financial capacity - Conflicts of interest | - Regulating the cost of private care - Regulation of the quality of care | - --- | - --- | - --- |
| 23 | Shehla, Z, et al, 2016 (23) | Eastern  Mediterranean Region | Role and contribution of the private sector in moving towards universal health coverage | - Private sector   (NGO)  Non-profit organizations  Non-state sector | - Health-service delivery - Maternal delivery or beds for chronic care services - Basic benefits package of curative and preventive primary health care - Chronic disease control, blood bank services and emergency transportation | - Undecided and uncertain regulation - Information on the technical process of care remains thin - Data gaps - Fragile programs - Insufficient information and lack of a database on private health sector - Private sector health care providers have no, or minimal role in developing national health policies - Lack of trust between the public and private sectors - Lack of, or poor, regulatory mechanisms - The role of the private health sector is not well defined - Capacities are poorly understood - Their contribution in preventive care and screening is not optimal and even practices and care delivery are not monitored - The range of services provided varies from one to another private care provider within the same country - Standards are questionable - Regulation is poor - Insufficient information about the financial burden to the users of these services - Out-of-pocket payment - Absence of a proper health management information system and a coordination mechanism - Balance tilting towards curative care - Lack of treatment protocols and guidelines to control and monitor the quality of services - Lack of commitment in low-income countries - Management information systems were inadequate | - Support by databases for private sector mapping - Accreditation tools - Adequate human resource - Budgetary support - Regulation, provision of consumer information, and purchasing of private health services - Build in adequate safety nets - Data-sharing on health services - Oversight on quality standards. - Collating, synthesizing and disseminating a list of “best buys” for public–private partnerships - Developing comprehensive and elastic frameworks and tools for evaluating public–private partnerships | - Poor quality of care in the public sector - Weak accessibility and affordability to comprehensive health services in the public sector - Patient safety - Particular emphasis on regulation, consumer information and purchasing/financing of private health services - Analysis of drivers and constraints in strengthening the role of the private health sector in establishing regulatory mechanisms and service provision - Proposing a framework that includes regulation, service provision and financing as a basis for the enhanced role of the private sector - Public sector spending on health is low - Purchase of private sector health services - Overburdening of public sector hospitals - Capacity constraints - Supplement the public sector which was damaged and weakened during conflict. - Fill coverage gaps in remote locations - Improve the functionality of existing service - Supplement ministry capacity - Priority - Explicit objectives | - Increase out-of-pocket expenditure | - Increased access to services - Improvement of infrastructure and availability of drugs, staff and supplies - Increasing the use of primary health care services - Enhanced equity by expanding service coverage levels for the poor - Cost curtailment of outing expensive infrastructure through using underutilized beds in the private sector - Attract patients from other countries - Expanded access - Improved quality - Increased cost efficiency - Enhanced equity |
| 24 | Stallworthy, G. Et al, 2014 (24) | Cross-Country | Roundtable discussion: what is the future role of  The private sector in health? | - Private sector   (NGO)  Non-profit organizations  Non-state sector | - Financing - Regulation - Provision | - Weakness government policies - Lack of protection from financial risk - Out of pocket | - Governments and donors must address public spending - Stimulate private investments and promote - Improvement in healthcare quality - Mobilize domestic resources - The review of fiscal policy (such as tariffs and import duties) - The improvement of regulatory frameworks through, quality accreditation and standards of care. - Improving access to finance for healthcare investments. - The development of different PPP models. - The engagement of the private sector, beyond health, in improving access to health services - **Cultural shift** - Alignment on the key gaps/priorities - Requires strong leadership to ensure that future | - Lack of investments - Low public spending - Limited inclusive mechanisms for pooling risks and resources. |  |  |
| 25 | Clarke, D. Et al. 2019 (25) | Cross-Country | The private sector and universal health coverage | - Private sector | - --- | - Governance and regulatory arrangements are not designed to effectively manage and coordinate mixed health systems - Threaten the UHC objectives of equity and quality - Abuse of market power (market skimming monopolistic behavior and predatory pricing) - Conflicts of interest - Regulatory capture - Incomplete information about the not-for-profit providers - Lack the governance tools to help align the activities of these providers with national systems and priorities - Private sector is heterogeneous | - Formulate domestic health goals and priorities - Multi Stakeholder involvement - Mix of legal and financial regulatory tools to manage - Regulatory mechanisms for licensing, certification and accreditation of health workers, medical products, services and facilities | - Health fiscal space constraints - Non-communicable disease burden increase - Demographic shifts including ageing - Population displacement - Political and economic instability - Offering access to greater service capacity - Managerial expertise - Higher quality of services, technology and innovation - Investment and funding. | - --- | - --- |
| 26 | WHO 2018 (26) | Cross-Country | The private sector,  Universal health coverage and  Primary health care | - Private sector   For-profit  Not-for-profit | - Direct provision of health services - Medicines and medical products - Financial products - Training for the health workforce - Information technology - Infrastructure - Support services | - Weak regulatory capacity - Conflicts of interests - The complexity and diversity of the private sector - Lack of conceptual clarity about the role of the private sector in health systems - Private sector is highly heterogeneous - Gaps in evidence - Normative gaps - Lack clear and comprehensive standards and frameworks | - Range of regulatory and financial policy tools to steer | - Fiscal space constraints arising from financial crises - Changes in disease burden - Demographic shifts - Population displacement - Cases of political and economic instability | - --- | - Access to greater service capacity - Greater responsiveness - Managerial expertise - Technology and innovation - Investment and funding |
| 27 | Grieve, A. &  Olivier, J. 2018 (27) | Ghana | Towards universal health coverage: a  Mixed-method study mapping the  Development of the faith-based non-profit  Sector in the Ghanaian health system | - Faith-based non-profit | - Provision of health services | - Lack of basic information - Inequitable - Financing and human resource crises - Lack of infrastructure in rural areas - Lack of transport systems typical in rural areas | - -- | - Inequitable geographical distribution of health services - Limited number of Public sector - Weak public system | - --- | - -- |
| 28 | Perumal-Pillay, V. A. &  Suleman, F. 2020 (28) | South Africa | Understanding the decision making  process of selection of medicines in the  private sector in South Africa – lessons for  low-middle income countries | - Private sector | - Medicines and medical products | - Affordability of essential medicines - Dearth of information on the processes for medicines selection for private sector - Lack of information to support pharmacy-economic evaluations of medicines - Conflict of interest - Low provision of information for decision-making - Alignment of formularies with SA EML - Pressure from pharmaceutical industry | - Include essential medicines in the benefit packages provided by public and private healthcare sectors - Formulary decision-making committees - Increased understanding of how decisions are taken to include/exclude medicines on private sector medical scheme formularies - Processes on monitoring and evaluation of medicines use by the private sector | - --- | - --- | - --- |
| 29 | Sieverding, M. et al, 2018 (29) | Ghana Kenya | We explore private providers' perceptions of and experiences with participation in two different social health insurance schemes in Sub-Saharan Africa the National Health Insurance Scheme (NHIS) in Ghana and the National Hospital Insurance Fund (NHIF) in Kenya. | - Private sector   For-profit private | - Participation in two social health insurance schemes and the National Hospital Insurance Fund | - Long delays in claims reimbursement - The lengthy and cumbersome accreditation process - Accreditation payment - Extensiveness of the checklist and inspection process - Administrative challenges - Tariff rates for medicines - Poor communication | - --- | - Fills gaps left in serving poor populations - Financial motivations - Strong client pressure | - --- | - --- |
| 30 | Onoka, C. A. et al, 2016 (30) | Nigeria | Growth of health maintenance organizations in Nigeria and the potential for a role in promoting universal coverage efforts | - Health maintenance organizations   (HMO) | - Health financing systems | - Risk selection by private insurers - Poor capacity to regulate private health insurance - Poor information | - Effective regulatory institutions - Policy guiding the public-private arrangement should be structured to promote the use of their infrastructural, financial and technical capacity to promote public health goals. | - Weak public systems | - --- | - --- |
| 31 | Meliala, A. et al, 2013 (31) | Indonesia | Addressing the unequal geographic distribution of specialist doctors in Indonesia: the role of the private sector and effectiveness of current regulations | - Private sector | - Human resource | - Less information - Maldistribution of specialist doctors - Dual practice - Limiting geographical access to health services in underserved areas - Uneven regional economic development - Persistent bureaucratic and administrative constraints following decentralization - Poor regulatory capacity - Low government investment in health - Lack of access to specialist doctors - Weaknesses in implementing regulations - Low levels of government salary - Rising income opportunities from the private sector | - Regulations limiting practice locations - Regulatory policies - Financial incentives | --- | --- | --- |
| **Manual search** | | | | | | | | | | |
| 32 | Doherty, Jane 2015 (32) | East and Southern Africa | Achieving  universal  health coverage  in East and Southern Africa:  What role for for‐profit  providers? | - For-profit private provision | - --- | - For-profit private sectors are not well documented - Low evidence exists on the health and equity outcomes of private services - High cost of private health care - Poor quality healthcare - Provides fragmented care - Undermines allocative efficiency - Fee for service payment - Fragment preventive, diagnostic and curative services - Brain drain from the public sector - Not coordinated - Little community participation - Over-servicing - High prices and inflation - Corruption and fraud - Poor at following national guidelines - Neglect critical services - Misuse pharmaceuticals - Difficult to work with regarding quality improvement programs - No quality control over illegal services - Serve only those able to pay - Concentrated in urban areas - Differentials in quality between public and private patients - Catastrophic financial impact on poor households - Weak regulatory systems - Limited competition between private providers - Poor enforcement of legislation - No formal policy on the private sector guided by public health objectives - Regulatory frameworks are usually patchy - Stakeholders conflict of interests - Regulatory authorities are fragmented and have poor capacity - Little information on the private sector | - Policy and regulatory responses need to take account of the different incentives inherent in these different situations - Strong regulatory and financing systems - Strengthening government capacity to develop, implement and monitor legislation and other regulations | - Weak public health systems - Considerable external support receives - Improve access to health care - Weak quality of services in both sector | - Private sector can worsen inequality - Poor quality, particularly where regulation is weak - Large private sector undermines the integrity and sustainability of the health system overall - Destabilizes the public system on which the poor depend | - --- |
| 33 | Titoria, R &  Mohandas, A. 2019 (33) | India | A glance on public private partnership: an opportunity for developing nations to achieve universal health coverage | - Private sector | - Service delivery - Human resource | - Huge population - Difficult land terrain - Unequal distribution of health care system - Socio-economic and cultural challenges - Conflict of interest - strong political commitment - out of pocket expenditure - inadequacies in public healthcare system | - Strategic purchasing of health services from private providers - Contractual agreement - Agreed objectives - Defined authority - Specified time period - Allocation of risk - Predetermined benchmarks - Performance linked payment to private sector - Defined authority of each partner - Commitment to agreed objectives - political will - Setting guidelines for effective monitoring and evaluation | - Human resource - Strengthen the health system by provision of financial and/or non-financial investment - Delivery of quality services - Increase in efficiency: upgrading the health infrastructure - Improved accessibility - Improved affordability - Risk sharing - Improved coverage of national health programs - Implementation of standard treatment guidelines - Scale-up successful operational models | - --- | - Easy access to services - Minimizing financial outgo government - Reliable stream of revenues for private sector |
| 34 | Shah, U  Thakur, H. 2018 (34) | India | Achieving Universal Health Coverage through Public Private Partnerships:  A Study of Trends in the Public Private Partnerships in India | - Private sector | - Create infrastructure - Healthcare delivery | - Access, coverage and availability of health services, for the underserved - Inadequate information - Risk sharing/ risk transfer mechanisms - Weak contract frameworks - Poor or limited support in providing - Occupancy certificate | - Policy modifications - Reconsider strategic purchasing - Building capacity within public health institutions - Provision of the framework for guidance | - Create additional and newer infrastructure - Upgrade and improve medical access and coverage - Limited financial resources - Limited capacity - Deliver better quality of services | - --- | - Lowering of financial burden - Ability to leverage managerial capabilities of the private sector |
| 35 | Jonty R, et al 2018 (35) | Low- and middle-income  countries | This report’s recommendations are aimed at the public and private sectors that can act together to achieve UHC, ultimately improving the condition of people across the world who still lack access to quality healthcare. | - Private sector | - Delivery of health services | - Purchasing poor-value care - Partnerships proving unsustainable - Profiteering actively - Out of pocket - Private investment remains only in the large cities - Financially challenges - Fragmentation by sector - Lack of standardization - Technical and resourcing challenge | - Alignment by providing investment, innovation and expertise - Health care standardization - Offering standardized service packages and prices that apply to all socioeconomic levels - Incentivizing evidence-based care - Providing assurances around scale and large volumes of patients - Imposing quality and reporting standards - Assess the readiness of the public and private sector for work together - Timely payments - Independent quality assessment - Price determination bodies - Workforce training. | - The technical and resourcing challenge | - --- | - --- |
| 36 | Gabrielle A. 2019 (36) | Cross-Country | Private Sector Accountability for Service Delivery in the Context of Universal Health Coverage | - Private sector | - Health service delivery | - Weak stewardship - Poor accountability within the public sector - Generally large private sector - Poorly documented - Very heterogeneous | - Requires organization of providers and formalization of engagement mechanisms | - --- | - --- | - --- |
| 37 | Hung, Y. W. et al, (37) | LMICs | Private health sector engagement in the journey towards Universal Health Coverage: Landscape Analysis | - Private sector | - Health service delivery | - Lack coordination - Limited information on private pharmacies - Challenges on implementation of regulation - Financial barriers - High out-of-pocket payments - Lack of quality control - Challenges of access to referral services - Changing demographics - Increase in non-communicable disease burden - Lack of awareness about the policy outside the Kampala district - Health officers unwilling to implement due to with conflicts regulations - Lacks resources - Lack of common understanding - Lack of systems and regulations for routine private sector reporting - Lack of integration in the public health information system - Cumbersome reporting process, - Lack of incentives - Lack of training provided to private sector staff in the reporting systems - Barriers to licensing: the cost of license and lack of qualifications to apply | - Administrative and bureaucratic regulation systems - Regulation on pricing of medical services and/or medications - Voucher scheme financing to reduce financial barriers - Effective governance of the private sector - Improving equity - Increasing access - Strengthening efficiency - Creating mixed health system that complements each other - Details the strategic priorities for the partnership - Active involvement of private health sector in national health strategic plans and establishment of formal dialogue mechanisms | - --- | - --- | - Improve health services provision - Increase of primary health care utilization - Improving equity - Increasing access - Strengthening efficiency - Creating mixed health system that complements each other |
| 38 | Joel S K. 2020 (38) | Cross-Country | Engaging the private health sector to advance Universal Health Coverage: A case study from the WHO Regional Office for Eastern Mediterranean Region | - Private sector | - --- | - --- | - Develop a policy framework, organizational systems, and financing strategies for engaging private health sector - Strategic purchasing - Facilitate and institutionalize private health sector engagement - Capacity-building - Improve the quality of services - Ensure regulatory mechanisms - Develop monitoring and reporting mechanisms | - Weak public sector | - --- | - --- |
| 39 | Hellowell, M &  O’Hanlon, B 2020 (39) | Cross-Country | Principles for Engaging the  Private Sector in Universal  Health Coverage | - Private sector | - --- | - Limited package of regulatory tools - Direct individual costs of service - Inadequate data - Lack the capacity to analyze data, particularly market data, on private sector activity - Private providers do not report to the ministry of health for fear of increased taxation - Lack of reciprocity - Regulatory barrier - Market condition barrier - Barrier of access to capital - Business skills barrier - Mistrust between the public and private sectors - Lack of understanding of the private health sector’s intentions | - Compulsory standards on premises - Certifying professional qualifications - Rely on strong governance - Pse approaches are defined by “problems” not “solutions” - Successful governance of the private sector requires good data - The private sector needs to be engaged in a meaningful dialogue - Using of tools of government for indirect governance of the private health sector - Economic regulation - Social regulation - facilitate voucher-style payment to clinics | - Build capacity | - --- | - --- |
| 40 | WHO (40) | WHO’s Eastern Mediterranean Region | Private sector engagement for advancing universal health coverage | - Private sector   (for profit health service) | - Provide ambulatory and hospital services - Infrastructure development - Production and supply of medicines and health technologies - Financing | - Lack of information from private providers - Poor regulate private sector - Sporadic partnerships between the public and private sectors - Inadequate organized - Weak policies and commitment, to regulate the in private health sector - Reluctant to invest in preventive care or in deprived areas - Weak regulatory systems - Weak mechanisms for monitoring the quality of health services - Limited and unreliable information on the distribution of the health workforce - Dual medical practice - Insufficient supply of essential medicines in the public sector - Irrational use of health technology - Limited government control of medicine promotion and advertising - Diagnostic facilities in the private health sector by high prices - Unneeded tests and lack of information about quality - Weak government regulatory oversight - Lack of regulation - Lack of capacity and resources - Lack of enforceable legal authority - Powerful lobbying by groups often undermines the regulatory framework | - Ensure regulatory mechanisms for health systems - Develop monitoring and reporting mechanisms - Improve the quality of services in the private health sector; - Develop a policy framework, organizational systems and financing strategies - Strategic purchasing - Facilitate and institutionalize private health sector engagement, including capacity-building | - The poor image and low quality of health care in the public sector - The absence of public health facilities in underserved areas - Large urban migration and the inability of the public health sector to cope with the increasing population in the urban fringes - Low government spending on health - Increased use of the private health sector - Higher out-of-pocket spending - Dual practice - High profits - Weak enforcement of the tax system - Harnessing resources | - --- | - Social and economic benefits |
| 41 | Wu, Runguo, et al, 2020 (41) | China | The purpose of study was synthesizing empirical evidence about PHI’s ability to play a role in financing China’s healthcare system and meet UHC principles | - Private health insurance | - Expand coverage - Improve access to care - Financial protection | - Out of pocket - Price control - Actuarial difficulties - Expanding its coverage prevalence - Meanwhile protecting equity - Lack of information - Limited phi depth coverage | - Define benefits package - Strong government regulations against voluntary enrolment and risk-pricing of phi | - Better allocate resources to the population - Improve weak administrative capability in the public sector - Solve limited public fiscal space for SHI - Out-of-pocket payment - Restricted governments financial capacity - Expansion of SHI and extend the depth and height of coverage | - Increased individual total health expenditure | - --- |
| 42 | UHC 2030, 2019 (42) | Cross-Country | Private Sector contributions  towards Universal Health Coverage | - Private sector   for profit private sector | - Health and related products, services, and innovation | - --- | - Leaving no one behind - Transparency and accountability for results - Evidence-based national health strategies and leadership - Making health everybody’s business - International cooperation based on mutual learning across countries - Contribute to efforts to raise the finance available for uhc - Engage in, champion and build capacities for relevant policy dialogue and partnerships with government and other stakeholders - Processes for structured and meaningful engagement of all partners - Government commitment to dialogue with all actors - National health strategies and plans that set clear, evidence-based policy objectives - Robust regulatory and legal system - Government stewardship of laws, standards, quality control and redress mechanisms - More and better investment in health, especially to strengthen health systems - Appropriate capacity and procedures to work with non-state actors - Knowledge and evidence on ‘what works’ for health systems and uhc | - Lacks access to essential health services - Contributions to training health workers | - --- | - --- |

1. Ejughemre UJ. Accelerated reforms in healthcare financing: the need to scale up private sector participation in Nigeria. International journal of health policy and management. 2014;2(1):13-9.

2. Roy B. Aspiring for universal health coverage through private care. Econ Polit Wkly. 2017;52(16):15-8.

3. Hallo De Wolf A, Toebes B. Assessing Private Sector Involvement in Health Care and Universal Health Coverage in Light of the Right to Health. Health and human rights. 2016;18(2):79-92.

4. Iyer V, Sidney K, Mehta R, Mavalankar D. Availability and provision of emergency obstetric care under a public-private partnership in three districts of Gujarat, India: lessons for Universal Health Coverage. BMJ global health. 2016;1(1):e000019.

5. Iyer V, Sidney K, Mehta R, Mavalankar D, De Costa A. Characteristics of private partners in Chiranjeevi Yojana, a public-private-partnership to promote institutional births in Gujarat, India – Lessons for universal health coverage. PloS one. 2017;12(10).

6. Cowley P, Chu A. Comparison of Private Sector Hospital Involvement for UHC in the Western Pacific Region. Health systems and reform. 2019;5(1):59-65.

7. Rao KD, Paina L, Ingabire MG, Shroff ZC. Contracting non-state providers for universal health coverage: learnings from Africa, Asia, and Eastern Europe. International journal for equity in health. 2018;17(1):127.

8. Maluka S. Contracting out non-state providers to provide primary healthcare services in Tanzania: Perceptions of stakeholders. International journal of health policy and management. 2018;7(10):910-8.

9. Maluka S, Chitama D, Dungumaro E, Masawe C, Rao K, Shroff Z. Contracting-out primary health care services in Tanzania towards UHC: how policy processes and context influence policy design and implementation. International journal for equity in health. 2018;17(1):118.

10. Olu O, Drameh-Avognon P, Asamoah-Odei E, Kasolo F, Valdez T, Kabaniha G, et al. Correction to: Community participation and private sector engagement are fundamental to achieving universal health coverage and health security in Africa: reflections from the second Africa health forum. BMC proceedings. 2019;13:11.

11. Lu JFR, Chiang TL. Developing an adequate supply of health services: Taiwan's path to Universal Health Coverage. Soc Sci Med. 2018;198:7-13.

12. Ssennyonjo A, Namakula J, Kasyaba R, Orach S, Bennett S, Ssengooba F. Government resource contributions to the private-not-for-profit sector in Uganda: evolution, adaptations and implications for universal health coverage. International journal for equity in health. 2018;17(1):130.

13. Wong EL, Yeoh EK, Chau PY, Yam CH, Cheung AW, Fung H. How shall we examine and learn about public-private partnerships (PPPs) in the health sector? Realist evaluation of PPPs in Hong Kong. Social science & medicine (1982). 2015;147:261-9.

14. Wadge H, Roy R, Sripathy A, Fontana G, Marti J, Darzi A. How to harness the private sector for universal health coverage. The Lancet. 2017;390(10090):e19-e20.

15. McPake B, Hanson K. Managing the public-private mix to achieve universal health coverage. Lancet (London, England). 2016;388(10044):622-30.

16. Shroff ZC, Rao KD, Bennett S, Paina L, Ingabire MG, Ghaffar A. Moving towards universal health coverage: engaging non-state providers. International journal for equity in health. 2018;17(1):135.

17. Nabyonga-Orem J, Nabukalu JB, Okuonzi SA. Partnership with private for-profit sector for universal health coverage in sub-Saharan Africa: opportunities and caveats. BMJ global health. 2019;4(Suppl 9):e001193.

18. Morgan R, Ensor T, Waters H. Performance of private sector health care: implications for universal health coverage. Lancet (London, England). 2016;388(10044):606-12.

19. Ota MOC, Kirigia DG, Asamoah-Odei E, Drameh-Avognon PS, Olu O, Malecela MN, et al. Proceedings of the first African Health Forum: effective partnerships and intersectoral collaborations are critical for attainment of Universal Health Coverage in Africa. BMC proceedings. 2018;12(Suppl 7):8.

20. Kumar R. Public-private partnerships for universal health coverage? The future of "free health" in Sri Lanka. Globalization and health. 2019;15.

21. Suchman L, Hart E, Montagu D. Public-private partnerships in practice: collaborating to improve health finance policy in Ghana and Kenya. Health policy and planning. 2018;33(7):777-85.

22. Tsevelvaanchig U, Narula IS, Gouda H, Hill PS. Regulating the for-profit private healthcare providers towards universal health coverage: A qualitative study of legal and organizational framework in Mongolia. Int J Health Plann Manage. 2018;33(1):185-201.

23. Organization WH. Role and contribution of the private sector in moving towards universal health coverage. 2016.

24. Stallworthy G, Boahene K, Ohiri K, Pamba A, Knezovich J. Roundtable discussion: What is the future role of the private sector in health? Globalization and health. 2014;10(1).

25. Clarke D, Doerr S, Hunter M, Schmets G, Soucat A, Paviza A. The private sector and universal health coverage. Bulletin of the World Health Organization. 2019;97(6):434-5.

26. Organization WH. The private sector, universal health coverage and primary health care. World Health Organization; 2018.

27. Grieve A, Olivier J. Towards universal health coverage: a mixed-method study mapping the development of the faith-based non-profit sector in the Ghanaian health system. International journal for equity in health. 2018;17(1):97.

28. Perumal-Pillay VA, Suleman F. Understanding the decision making process of selection of medicines in the private sector in South Africa - lessons for low-middle income countries. Journal of pharmaceutical policy and practice. 2020;13:17.

29. Sieverding M, Onyango C, Suchman L. Private healthcare provider experiences with social health insurance schemes: Findings from a qualitative study in Ghana and Kenya. PloS one. 2018;13(2).

30. Onoka CA, Hanson K, Mills A. Growth of health maintenance organisations in Nigeria and the potential for a role in promoting universal coverage efforts. Social science & medicine (1982). 2016;162:11-20.

31. Meliala A, Hort K, Trisnantoro L. Addressing the unequal geographic distribution of specialist doctors in indonesia: the role of the private sector and effectiveness of current regulations. Social science & medicine (1982). 2013;82:30-4.

32. Doherty J, editor Achieving universal health coverage in East and southern Africa: what role for for-profit providers. International Conference on Public Policy, Panel session T03P13; 2015.

33. Titoria R, Mohandas A. A glance on public private partnership: an opportunity for developing nations to achieve universal health coverage. International Journal of Community Medicine and Public Health. 2019;6(3):1353.

34. Shah U, Thakur H. Achieving Universal Health Coverage through Public Private Partnerships: A Study of Trends in the Public Private Partnerships in India. International Journal of Health Sciences & Research. 2018;8(10).

35. Roland J, Bhattacharya-Craven A, Hardesty C, Fitzgerald E, Varma N, Aufegger L, et al. Healthy Returns: The Role of Private Providers in Delivering Universal Health Coverage. Doha, Qatar: World Innovation Summit for Health. 2018.

36. Appleford G. Private Sector Accountability for Service Delivery in the Context of Universal Health Coverage. Geneva: World Health Organization. 2019.

37. Hung YW, Klinton J, Eldridge C. Private health sector engagement in the journey towards Universal Health Coverage: Landscape Analysis. Geneva: World Health Organization. 2020.

38. Klinton JS. Engaging the private health sector to advance Universal Health Coverage: A case study from the WHO Regional Office for Eastern Mediterranean Region. Geneva: World Health Organization. 2020.

39. Hellowell M, O’Hanlon B. Principles for Engaging the Private Sector in Universal Health Coverage: A Background Report for The Advisory Group on the Governance of the Private Sector for UHC. Geneva: World Health Organization. 2020.

40. Organization WH. Private sector engagement for advancing universal health coverage. World Health Organization. 2018.

41. Wu R, Li N, Ercia A. The effects of private health insurance on universal health coverage objectives in China: a systematic literature review. International journal of environmental research and public health. 2020;17(6):2049.

42. UHC2030. Private Sector contributions towards Universal Health Coverage UHC2030 Private Sector Constituency Statement. <https://wwwuhc2030org/fileadmin/uploads/uhc2030/Documents/Key_Issues/Private_Sector/UHC2030_Private_Sector_Constituency_Joint_Statement_on_UHC_FINALpdf>. 2019.
